# Spontaneous Coronary Artery Dissection following Topical Hormone Replacement Therapy

**DOI:** 10.1155/2012/524508

**Published:** 2012-08-15

**Authors:** Alexander L. Pan, David Fergusson, Robert Hong, Ramy A. Badawi

**Affiliations:** Division of Cardiology, The John A. Burns School of Medicine, University of Hawaii, Queens Medical Center, Honolulu, HI 96813, USA

## Abstract

Spontaneous coronary artery dissection is a rare condition, usually presenting as an acute coronary syndrome, and is often seen in states associated with high systemic estrogen levels such as pregnancy or oral contraceptive use. While topical hormonal replacement therapy may result in increased estrogen levels similar to those documented with oral contraceptive use, there are no reported cases of spontaneous coronary dissection with topical hormonal replacement therapy. We describe a 53-year-old female who developed two spontaneous coronary dissections while on topical hormonal replacement therapy. The patient had no other risk factors for coronary dissection. After withdrawal from topical hormonal therapy, our patient has done well and has not had recurrent coronary artery dissections over a one-year follow-up period. The potential contributory role of topical hormonal therapy as a cause of spontaneous coronary dissection should be recognized.

## 1. Introduction

Spontaneous coronary artery dissection (SCAD) is a rare condition, usually presenting as an acute coronary syndrome (ACS) in young or middle-aged women, and is often associated with pregnancy or oral contraceptives. While topical hormonal therapy may result in increased estrogen levels similar to those documented with oral contraceptive use, SCAD occurring after topical hormone therapy has not been reported.

We report a case of recurrent spontaneous coronary artery dissection in a 53-year-old woman who had been treated with topical hormone replacement therapy for 4 years.

## 2. Case Report

**Figure 1:**
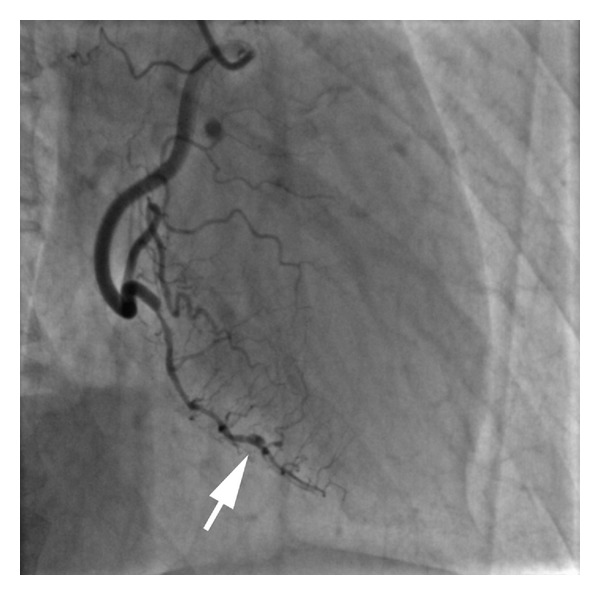
Initial study: right coronary injection showing irregular narrowing in the posterior descending artery, extending antegrade and retrograde from the apparent point of dissection (arrow).

A 53-year-old postmenopausal female, with a history of treated hypothyroidism, presented to our medical center with protracted severe left-sided substernal rest chest discomfort. The patient had been using topical hormonal replacement therapy with 2 compounded creams: Biest (80 : 20 of estriol : estradiol) 5 mg/day and topical progesterone 125 mg/day for the previous 4 years. An initial electrocardiogram demonstrated inverted T waves in the inferolateral leads but no ST elevations. The patient was treated with nitrates with the relief of her symptoms. Cardiac biomarkers were obtained and found to be abnormal with an increased troponin I—level of 0.16 ng/mL (normal range <0.05 ng/mL). The patient was treated with aspirin, low molecular weight heparin, and intravenous nitroglycerin. An echocardiogram documented a normal left ventricular systolic function and no segmental wall motion abnormalities. Coronary angiography revealed spontaneous coronary dissection in the posterior descending branch of the right coronary artery. An apparent point of dissection was identified relatively distally, with contrast entering the false lumen beyond this point, and persisting with delayed contrast washout (Figure 1). Given the distal location of the dissection and clinical stability, a conservative medical treatment without coronary interventions was chosen.

**Figure 2:**
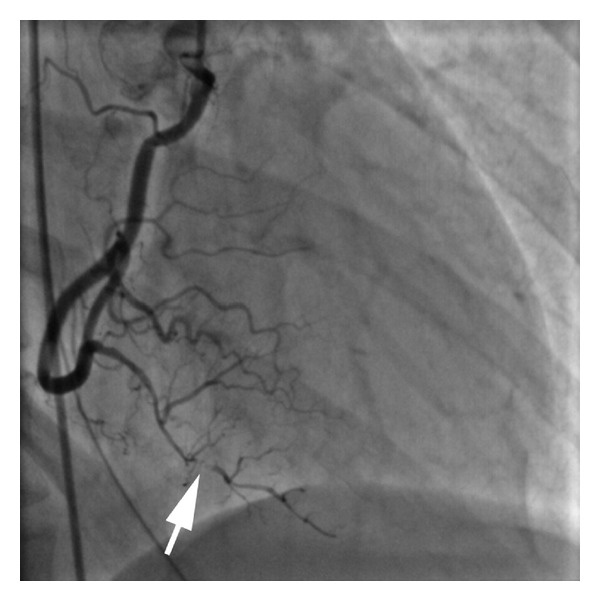
Second study showing an intraluminal filling defect (arrow) proximal to the original site of dissection.

Thyroid stimulating hormone level, erythrocyte sedimentation rate, and antinuclear antibody levels were unremarkable. Urine toxicology testing did not demonstrate the presence of cocaine or amphetamines. Serial cardiac biomarkers were noted to increase with a peak troponin I level of 1.31 ng/mL. The patient was treated with aspirin and metoprolol and discharged. One day after discharge, the patient developed recurrent severe chest discomfort that radiated to her left arm and ventricular fibrillation. The patient was resuscitated. A repeat electrocardiogram demonstrated 3 millimeter ST segment elevations in the inferior leads. She underwent emergent repeat coronary angiography immediately after resuscitation. She was documented to have a small intraluminal filling defect proximal to the apparent original site of dissection, thought to represent a thrombus (Figure 2) or recurrent dissection. The patient was documented to have impaired left ventricular function with extensive apical ballooning. Postresuscitative course was complicated by hypotension requiring vasopressor and transient intraaortic balloon counterpulsation support. Repeat assessments of cardiac biomarkers documented limited cardiac injury with a peak troponin I level of 2.02 ng/mL. Serial electrocardiograms demonstrated resolution of the inferior ST elevations and no Q waves, and she was weaned off vasopressors and intraaortic counterpulsation support. Topical hormonal therapy was discontinued, and she has been followed for 12 months without recurrent cardiac events.

## 3. Discussion

Spontaneous coronary dissection (SCAD) is a rare cause of acute coronary syndrome. Since its first description in 1931 [1], over 440 cases has been reported [2]. The incidence of SCAD has been estimated to be 0.07% to 1.1% [2, 3]. SCAD typically affects young or middle-aged females. The mean age of presentation is 42 (range 17 to 82) [2], with females accounting for *t* 70% of cases. A peripartum association with SCAD has been noted in 30% of the reported cases [4, 5].

Spontaneous coronary dissection most commonly involves the left anterior descending artery but involvement of all coronary arteries and multivessel dissection have been described [2].

Multiple etiologic factors for SCAD have been postulated. A common mechanism for all proposed causes is a mismatch between increased hemodynamic shear forces on the coronary arterial wall and/or decreased endovascular integrity. 

SCAD may be precipitated by vigorous exercise [6], cocaine use [7], and prolonged sneezing or retching. These actions are proposed to increase shear stress in the coronary arterial wall. Impaired endovascular integrity may also contribute to a propensity for SCAD. SCAD is reported in diseases associated with abnormal vascular integrity such as Ehlers-Danlos syndrome, Marphan syndrome, and idiopathic cystic medial degeneration. Vasculitis associated with systemic lupus erythematosus and polyarteritis nodosa may also be associated with SCAD.

The reasons for SCAD in states of increased estrogen or female hormonal action are less clear. During the pregnancy and the peripartum period, both increased arterial wall stress and impaired endovascular integrity have been proposed to contribute to SCAD. Increased wall stress may be noted in pregnancy because of normal physiologic changes such as increased total blood volume and cardiac output. Vasomotor changes associated with labor may also contribute to increased wall stress. In addition to increased wall stress, pregnancy or hormonally induced endovascular changes may contribute to a propensity for SCAD. Changes in elastic fiber arrangement and mucopolysaccharide content [4, 8], associated with hormonal states, likely contribute to impaired coronary vascular integrity. In over 50% of autopsied peripartum cases, periadventitial eosinophilic infiltrates have been noted. These infiltrates may be caused by lytic proteases that contribute to the disruption of the normal arterial wall [9].

SCAD following use of oral contraceptives has also been well described. The physiopathology of SCAD with oral contraceptive use is felt to be similar to that associated with pregnancy. Azam et al. [10] described a patient with SCAD who had been using ethinyl estradiol 30 mcg/day and levonorgestrel 150 mcg/day. This hormonal dosing was similar to a case described by Censori et al. [11], in whom a patient presented both with SCAD and spontaneous right extracranial internal carotid dissection. Zehir et al. [12] described SCAD in patient using a 3rd generation low-dose oral contraceptive (ethinyl estradiol 30 mcg and drospirenone 3 mg). The duration of oral contraceptives usage in the above cases ranged between 3 and 17 years prior to the index event [8, 10–12]. To our knowledge, there are no reported cases of topical hormone replacement therapy associated with SCAD. Our patient had been using 2 compounded creams: Biest (80 : 20 of estriol : estradiol) 5 mg/day and topical progresterone 125 mg/day for the previous 4 years. The absorption profile and high bioavailability of these would be expected to produce effects comparable to those of previously reported oral contraceptives.

## 4. Conclusion

Pregnancy and oral contraceptives are well known to be associated with SCAD. We report a case of topical hormone replacement therapy associated with SCAD. Recognition of the potential contributory role of hormonal therapy regardless of mode of administration in the pathogenesis of spontaneous coronary artery dissection is important for the clinician.
